# Physicochemical Characteristics, Microstructure and Health Promoting Properties of Green Banana Flour

**DOI:** 10.3390/foods10122894

**Published:** 2021-11-23

**Authors:** Minenhle Khoza, Eugenie Kayitesi, Bhekisisa C. Dlamini

**Affiliations:** 1 Department of Biotechnology and Food Technology, Faculty of Science, DFC Campus, University of Johannesburg, Doornfontein, Johannesburg 2028, South Africa; bcdlamini@uj.ac.za; 2 Department of Consumer and Food Sciences, University of Pretoria, Pretoria 0028, South Africa; eugenie.kayitesi@up.ac.za

**Keywords:** green banana flour, functional characteristics, in vitro starch digestibility, antioxidant activity

## Abstract

This study aimed to investigate the proximate composition, mineral content, functional properties, molecular structure, in vitro starch digestibility, total phenolic content (TPC), total flavonoid content (TFC) and antioxidant activity (DPPH, FRAP) of green banana flour (GBF) cultivars grown in South Africa. With proximate composition, Finger Rose and Pisang Awak had the highest protein (4.33 g/100 g) and fat (0.85 g/100 g) content, respectively. The highest ash content (3.50 g/100 g) occurred with both Grand Naine and FHIA-01 cultivars. Potassium and copper were the most abundant and least minerals, respectively. Pisang Awak cultivar had the highest water absorption capacity (67.11%), while Du Roi had the highest swelling power (0.83 g/g) at 90 °C. Scanning electron microscopy (SEM) images revealed that starch granules from all GBF cultivars were irregular in shape and they had dense surfaces with debris. All the GBF cultivars had similar diffraction patterns with prominent peaks from 15°–24° diffraction angles. The resistant starch (RS) and amylose content of the FHIA-01 cultivar indicates that the GBF has the potential to lower risks of type 2 diabetes and obesity. The highest TPC, TFC and antioxidant activity occurred with the Grande Naine cultivar. Based on their functional characteristics, the Grand Naine and FHIA-01 GBF cultivars could potentially be used as raw materials for bakery products as well as for the fortification of snacks.

## 1. Introduction

The diabetes endemic continues to increase associated with obesity, inactive lifestyles and high-energy diets [1]. These diseases are the leading cause of demise and disability worldwide. Globally 366 million people suffer from diabetes. The International Diabetes Federation predicts this number to rise to 552 million by 2030 [2]. The prevalence of diabetes in South Africa has remarkably increased over the past two decades, making South Africa one of the countries with the highest predictable upsurge in diabetes for the next twenty-five years [3,4]. Epidemiological studies show that frequent consumption of high glycemic index (GI) food may lead to a high risk of obesity and type II diabetes. As one of the solutions, studies have indicated that obesity and type II diabetes can be prevented by eating low GI foods such as green banana flour (GBF) [5]. Further, studies have revealed that adequate consumption of fruits and vegetables is vital for reducing the burden of heart diseases and diabetes, possibly due to their relatively high dietary fiber, resistant starch, antioxidants and bioactive compounds contained in these foods [1,6,7]. As a result, there has been an intensive development of secondary food products made from fruits and vegetables as sources of dietary fiber and indigestible starches, with more focus on developing new products. The development of such food products allows the consumer to have permanent access to the nutritional benefits of fruit and vegetable products, in spite of their seasonality, and thus healthy food can be made available throughout the year [8].

The use of green banana flour is important as an alternative raw material for the processing of healthy functional products. According to Kumar et al. [9], green banana flour has the following composition: 52.7–54.2% Resistant starch (RS); 1.81% Total Soluble Solids (TSS), 76.77% Total Starch and 14–17% non-starch polysaccharides. Similar to dietary fiber, starch from green banana flour is not digestible in the small intestine; hence, it is fermented in the colon by gut flora [10]. When RS reaches the colon, it is used as a substrate for microbial fermentation, and it may lead to the production of short-chain fatty acids (butyrate, propionate and acetate), carbon dioxide, hydrogen and methane [11]. Each type of short-chain fatty acid has impacts on health. Butyrate is well-known for health enhancement as it plays a vital role in human gut health, including: decreasing inflammation, reducing the risk for colon cancer and enhancing gut barrier functions [12]. The lower digestibility of RS leads to a reduced release of blood glucose. This has been shown to have a reduction in leptin and post-prandial glucose reactions in people after the consumption of food products with a high RS content [13]. Recently, food products with a low glycemic index are highly favored by consumers due to their resistance to glucoamylase and α-amylase. Further, their digestion rate in the gut is relatively low due to the presence of resistance starch, thus causing reduced energy intake by gastral cells [14,15]. The aim of the study was to investigate the proximate composition, mineral content, functional, molecular, microstructure, TPC, TFC and antioxidant activity of green banana flour cultivars grown in South Africa.

## 2. Materials and Methods

### 2.1. Materials

Flour from five green banana cultivars, namely Grande Naine, Pisang Awak, Finger Rose, FHIA-01 and Du Roi, was kindly provided by the Agricultural Research Counsel (ARC) Tropical and Subtropical Crops, Nelspruit, Mbombela in South African. All reagents were analytical grade, Trolox, Folin–Ciocalteu reagent, gallic acid and quercetin were purchased from Sigma-Aldrich Pty. Ltd. (Johannesburg, South Africa). The resistant starch assay kit and the amylose/amylopectin kit were purchased from Megazyme Ltd. (Johannesburg, South Africa).

### 2.2. Preparation of Banana Starch

A water-alkaline extraction method was used to prepare banana starch, as described by Jiang et al. [16], with a few changes. Briefly, GBF (100 g) was macerated in distilled water (1 L) for 20 min at a low speed, then sieved through 100-mesh screens. The collected milk was centrifuged at 4000× *g* for 10 min to remove soluble fiber, and then 1 L NaOH solution (0.2%, *w*/*v*) was added to the sediment. The starch sediment was mixed with water and stirred for 5 min before resting for 2 h. Thereafter, the sediment was again suspended in water and allowed to settle. This was repeated until the wash water reached a neutral pH. The resultant material was then dried at 45 °C for 24 h. The desiccated starch was pulverized and passed through a 100 µm sieve.

### 2.3. Proximate Composition of Green Banana Flour

The moisture content was assayed using a vacuum oven dryer at 60 °C for 16 h using 2–3 g of sample, according to Rodriguez-Jimenez et al. [17]. A furnace was used to measure ash content using a method described by [9]. Soxhlet extraction was used for total fat content [14]. For protein content, the Kjeldahl method was followed, as demonstrated by Kumar et al. [9]. The carbohydrate percentage was calculated using the formula below.

Carbohydrate (g) = 100 − (protein (g) + moisture content (g) + lipid (g) + ash (g))

### 2.4. Mineral Composition of Green Banana Flour

Mineral analysis was conducted following a method by Jakavula et al. [18]. Briefly, the sample was digested using ultra-pure HNO3 on a microwave-accelerated reaction system (CEM, Matthews, NC, USA). This was conducted at high temperature and pressure for the extraction of acid-extractable elements with the sample material. After that, deionized water was added (50 mL), followed by analyses of the sample by ICP-OES (Thermo Scientific, Basingstoke, UK).

### 2.5. Characterization of Functional Properties of Green Banana Flour

#### 2.5.1. Water Absorption Capacity (WAC)

The WAC of GBF was determined using the method described by Kumar et al. [9], with some modifications. Precisely, 0.5 g flour sample was weighed into 50 mL centrifuge tubes followed by the addition of 5 mL distilled water. The suspensions were vortexed and rested for 1 h at room temperature (26 ± 2 °C). Thereafter, they were centrifuged at 3000 rpm for 30 min at 25 °C. The WAC was expressed as mL of water absorbed per gram of flour.

#### 2.5.2. Water Solubility Index and Swelling Power

The water solubility index and swelling power were determined following the method detailed by Kumar et al. [9]. Green banana flour (0.2 g) was mixed with distilled water (5 mL) for 30 s using a vortex. After that, the mixture was heated at 50 °C, 70 °C and 90 °C for 20 min, followed by cooling and centrifugation at 3000 rpm for 10 min. The supernatant was evaporated at 105 °C for 16 h in an oven. The solubility index was calculated as the ratio of the mass of dried supernatant to the mass of the flour expressed in percentage (g/100 g DW). After centrifugation, the filtrate was also weighed to obtain the swelling power.

### 2.6. Microstructure Analysis of Green Banana Flour

#### 2.6.1. Scanning Electron Microscopy (SEM)

A scanning electron microscope (SEM-EDX) (JEOL, JSM 7500F) was used to study the microstructures of the banana starch granules. The GBF starch samples were placed on aluminum cylinders that had a double-sided tape followed by coating with carbon. The acceleration voltage was 10.00 kV, as previously described by Maziya et al. [19]. An electron beam with the resolution set at a particle size of 20–200 μm was used to view the microstructure of the samples.

#### 2.6.2. X-Ray Diffraction (XRD)

The XRD analysis of the GBF samples was determined using Philips X’Pert XRD equipment (Malvern PANalytical, Almelo, The Netherlands). The power source was set at 40 kV and 40 mA power with a scanning interval of 5°/min. The scanning range was 2θ = 5° to 90° [19].

### 2.7. Molecular Structure Analysis of Green Banana Cultivars

#### 2.7.1. Fourier Transform Infrared (ATR-FTIR) Spectroscopy

The ATR-FTIR spectra of GBF samples were measured using a 4000 FTIR spectrophotometer (JASCO, South Africa). The functional groups of the isolated compound were detected by ATR (JASCO, South Africa) with a diamond crystal plate with a scan rate of 16 runs per scan at a resolution of 4 cm^−1^ in wavenumbers from 500 to 4000 cm^−1^ [20].

#### 2.7.2. Determination of Rapidly Digestible, Slow Digestible, Resistant, and Total Starch Contents of Green Banana Flour

The determination of rapidly digestible, slow digestible, resistant, and total starch contents of green banana starch was carried out using a Megazyme Resistant Starch Assay Kit (Megazyme Ltd., Johannesburg, SA). Briefly, the method involved incubating the GBF sample (80 mg) in a mixture of enzymes (pancreatic α-amylase and amyloglucosidase) in maleate buffer (pH 6.0) (K-RNTDF; AOAC Method 2017.16) [21].

#### 2.7.3. Amylose and Amylopectin

A commercial amylopectin/amylose kit (Megazyme Ltd., Johannesburg, South Africa) was used to quantify amylose content. The principle of the method involves the separation of amylopectin and amylose. Thereafter, amylopectin is precipitated with concanavalin-A (Con A), followed by centrifugation to eliminate it Jiang et al. [16].

### 2.8. Total Phenols, Flavonoids Content and Antioxidant Properties

#### 2.8.1. Total Phenolic Content (TPC)

The TPC was determined using the Folin–Ciocalteu assay method according to the procedure outlined by Blainski et al. [22]. Briefly, one gram of GBF was mixed with a 25 mL mixture of methanol and water (*v*/*v*, 20:5, respectively) followed by incubation at 37 °C for 4 h with shaking. The mixture was then centrifuged (4000× *g* for 10 min). Thereafter, the supernatant was mixed with 500 µL deionized water in a test tube with 30 µL standard/extracts and 50 µL Folin–Ciocalteu reagent (Sigma-Aldrich, Johannesburg, South Africa). This was followed by the addition of 245 µL deionized water and 200 µL of Na_2_CO_3_. The sample mixture was then incubated at 27 °C for 30 min, and a microplate reader was used to measure the absorbance (750 nm). Gallic acid was used as standard, and the results were expressed as mg gallic acid equivalent (GAE)/g dry weight using the standard curve (R^2^ = 0.9982).

#### 2.8.2. Total Flavonoid Content (TFC)

The TFC was determined following the method described by Jabri-Karoui et al. [23]. Briefly, where Quercetin (Sigma-Aldrich, Johannesburg, South Africa) was used as a standard. An aliquot of 30 µL of each extract or standard {Quercetin (Sigma-Aldrich, Johannesburg, South Africa)} was mixed with 20 µL of 10% AlCl_3_ and 20 µL of 2.5% NaNO_3_. After 5 min, 100 µL of NaOH solution was added to the mixture. From the mixture using a micropipette, 200 µL was pipetted into a microplate (96 well). A microplate reader was used to measure the absorbance (450 nm). The TPC was expressed as mg QE/mg dry weight using the standard curve (R^2^ = 0.9991).

#### 2.8.3. Antioxidant Activity

The antioxidant activity of GBF samples was determined through the use of the ferric-reducing-ability-plasma (FRAP) with slight adjustment as described by Hofmann et al. [24]. The method involved the use of 2,2-diphenyl-1-picrylhydrazyl radical (DPPH) to assay the scavenging activity [25] of green banana flour. Trolox stock was used as standard, and the absorbance was read at 517 nm. The results were expressed as µM Trolox equivalent per 100 g of green banana flour (d.w.).

### 2.9. Statistical Analysis

One-way analysis of variance (ANOVA) was performed using the Statistica statistical software (Version 13.0/September 2015) for data analysis. The significant difference between the samples was determined at 95% (*p* ≤ 0.05). The results were shown as means ± standard deviation. The contrast of mean values was analyzed by Fisher Least Significant Difference (LSD) tests. All the experiments were performed in triplicates.

## 3. Results and Discussion

### 3.1. Proximate Composition of Green Banana Flour

The proximate composition of green banana flour (GBF) grown in South Africa is shown in Table 1. The GBF cultivars varied significantly in moisture content, with the Grand Naine and Finger Rose cultivars showing the same and highest (10.50 g/100 g d.w.) moisture content, while the FHIA-01 cultivar showed the lowest (9.40 g/100 g d.w.) moisture content. The moisture content of GBF cultivars reported in the current study is within the range generally reported in the literature for unripe/green banana flour. Similar to the findings of the present study, Kumar et al. [9] recorded 8.59% moisture content on green Grand Naine banana flour. Utrilla-Coello et al. [26] reported 7.03% moisture content for unripe Enano cultivar and 8.96% for unripe Valery banana cultivar. The moisture content of flour products is critical as it can have an influence on both the physical and chemical properties of foods. It can affect the shelf life and stability of foods since high moisture tends to cause changes in chemical, biochemical and textural properties as well as promoting microbial growth [9]. The relatively low moisture content of the GBF of this study suggests that it could be stable and may have an extended shelf life.

The abundance of minerals in GBF has made it a valuable fruit. In this study, the total ash content statistically (*p* ≤ 0.05) varied from 2.46–3.50 g/100 g d.w., with the Grand Naine cultivar recording the highest ash content. Campuzano et al. [27] reported an ash content of 2.61 g/100 g d.w. in GBF (Cavendish), and this is within the range of ash content found in the present study. Elsewhere, Kumar et al. [9] reported ash content of 2.06 and 2.50% for unripe Grand Naine and Nendran flours, respectively, with the former cultivar relatively lower in ash content than that of the current study. The variation in ash content could possibly be an indication of differences in mineral contents of the GBF cultivars, which can be attributed to agricultural practices and climate change [28] Further, the variations could also be linked to the differences in the type of soil under which they were grown. In general, the ash content of food is associated with a high presence of minerals such as calcium, magnesium, potassium and phosphorus [29].

The protein content of the five GBF cultivars significantly differed (*p* ≤ 0.05). High protein content occurred with the Finger Rose cultivar (4.33%), while the Grand Naine cultivar had the lowest protein content (3.60%). The Grand Naine protein content in this study was comparable with the protein content (3.53%) for the Grand Naine cultivar reported by Kumar et al. [9] on GBF from dessert and plantain banana (*Musa* spp.). In the study reported by Ferreira et al. [30] a protein Ferreira content of 1.94% was determined for green banana flour. Elsewhere, Ferreira et al. [31] reported similar protein content (1.89%) in unpeeled green banana flour. Bi et al. reported a protein content of 2.90% for Pisang Awak, which is significantly lower than the 4.12% found in the present study. The observed variation in results could be due to differences in the soil type and the stage of growth of the fruit [32].

The Pisang Awak cultivar had the highest fat content (0.85 g/100 g d.w.) with the lowest fat content recorded for the Du Roi cultivar (0.42 g/100 g d.w.) (Table 1). The fat content results reported here are within the range (0.92–0.93 g/100 g d.w.) reported by Khoozani et al. [29] in green Cavendish flour. According to Ye et al. [33] low-fat content reduces the extent of starch granule swelling. The low-fat content of banana flour creates an environment that is not suitable for oxidation reactions, resulting in extended shelf life. In fact, it reportedly decreases the risk of lipid oxidation which may result in extended shelf life. Variations in the chemical composition of banana cultivars are associated with various factors, such as regional climate, agronomic methods, harvesting conditions, among others [34]. However, the differences that occurred in the current study are attributed to cultivar variation since the cultivars were grown under the same environmental condition.

### 3.2. Mineral Composition of Green Banana Flour

The mineral profiles of GBF used in this study are shown in Table 2. In general, potassium (K) was the most abundant (290.95–1033.25 mg/100 g) mineral, while copper (Cu) was the least abundant (0.25–0.50 mg/100 g) mineral among the five GBF cultivars. FHIA-01 recorded the highest amount of K (1033.25 mg/100 g). The results attained from this study confirm that some banana cultivars (FHIA-01, Grande Naine and Finger Rose, respectively) cultivated in Mpumalanga Province, South Africa, appear to be an excellent source of K. The K level in this study was within the range (9117.32–14,746.73 mg/kg) reported by Tasnim et al. [35] in unripe banana flour cultivars attained from domestic and commercial farms in Limpopo, South Africa. With magnesium, FHIA-01 had the highest concentration (118.15 mg/100 g), while the Finger Rose cultivar had the lowest (82.10 mg/100 g). The research has indicated that the risk of diabetes can be reduced by consuming a high-Mg diet, and this has been associated with the role that Mg plays in glucose metabolism. Phosphorus (P) was within the range of 31.72–99.25 mg/kg for all GBF cultivars. There were significant differences (*p* ≤ *0.05*) between the content of sulfur (S) amongst the five GBF cultivars. With essential minerals, calcium (Ca) was the least abundant mineral with the lowest concentration observed in the Finger Rose (8.70 mg/100 g) cultivar.

As expected, the overall concentration of macro-minerals was higher than that of trace minerals. The Grande Naine cultivar recorded the highest (2.88 mg/100 g) concentration of iron (Fe), while Finger Rose recorded the lowest (1.50 mg/100 g). The concentration of Fe and Zn reported in the present study was lower than that reported by Ferreira et al. [36] who reported an Fe concentration of between 8.15–33.72 mg/kg and a Zn concentration of 3.55–7.78 mg/kg for commercial as well as uncommercial unripe banana flour. The Fe concentration of GBF cultivars in this study were higher than that reported by Pessoa et al. [37] (22.5–62.8 mg/kg) in GBF cultivars from Brazil. According to Freeland-Graves et al. [38], food such as beans, bovine liver and seafood are known to be good sources of iron; hence, they are termed “iron-rich foods”. Interestingly, the concentration of Fe in GBF cultivars investigated here were similar or higher than that of the aforementioned food products. This means the GBF cultivars could potentially be used as a good source of Fe in foods. The copper (Cu) concentration significantly (*p* < 0.05) varied from 0.25–5.0 mg/100 g, with the Grande Naine cultivar showing the highest concentration. The concentration of manganese (Mn) ranged from 0.48–3.2 mg/100 g, and the highest and lowest concentrations were observed in Grande Naine and Du Rio cultivars, respectively. Interestingly, the mineral composition of the soil has been reported to influence the mineral content of food crops [38]. Further, the pH as well as the amount of organic matter in the soil may also influence the mineral content. Other studies have reported similar influence with different agricultural practices and climate change [36,39].

### 3.3. Characterization of Functional Properties Banana Flours

#### 3.3.1. Water Absorption Capacity (WAC) of Green Banana Flour Cultivars

The WAC indicates the volume occupied by starch granules after swelling in excess of water [34]. It is affected by how much native starch granules have disintegrated. In addition, it is influenced by the physical state of starch, dietary fiber and proteins [40]. In this study, the WAC varied with the type of cultivar (Table 3). Pisang Awak had the highest WAC (67.11%), while the least WAC (40.00%) was observed with the Finger Rose cultivar. Campuzano et al. [27] reported an almost similar WAC range (48.50–70.00%) to that of the current study in GBF at different stages of ripeness. Pereira et al. [40] reported 80.00% WAC for green banana flour. The hydrophilic sites in starch chains allow for interaction with water through hydrogen bonding. The high WAC observed with Pisang Awak GBF here suggests that it could be suitable for baking. It must also be noted that WAC influences gelatinization through available water, and thus, a lower WAC is desirable for a thinner consistency [29].

#### 3.3.2. The Water Solubility and Swelling Power of Green Banana Flour Cultivars

The solubility and swelling power are parameters used to investigate the quality of starch granules. The solubility index is linked to the soluble solid contents in flour, whereas swelling power is a measure of the retention of starch granule integrity when subjected to high cooking temperatures [35,41]. Here, swelling patterns differed amongst the GBF cultivars. The swelling power increased with an increase in temperature with all cultivars (Table 4). Flour from the Du Roi cultivar had the highest swelling power (0.83 g/g) at 90 °C, while the FHIA-01 cultivar had the lowest swelling power (0.52 g/g) compared to all the other cultivars at the same temperature. The swelling of starch granules follows different stages. First, thermal energy is attained with heating, and this helps to loosen the intra-granular links of starch granules. When the temperature exceeds 70–80 °C, more rapid swelling of starch granules occurs possibly due to intermolecular hydrogen bonds breaking in the amorphous area [42,43]. The current results suggest that swelling of starch granules and high water penetration are attained at high temperatures for the investigated GBF cultivars. The solubility index and swelling power denote the range of interaction within the crystalline (amylose) and amorphous (amylopectin) regions of the starch molecule, along with the degree of branching and the length of branches [29]. Therefore, an increase in solubility index and swelling power cause gelatinization, which is the foundation for making pre-gelatinized starch. According to Khoozani et al. [29] significant differences in swelling may be attributed to low solubility, restricted swelling, the amylose content of flour and slight retrogradation (a reaction that takes place in gelatinized starch, when disaggregated amylopectin and amylose chains reassociate to form more ordered structures). Comparably, low solubility coupled with low swelling power indicates a more well-arranged, denser and strongly bonded granule structure. Another factor that can be attributed to the differences in swelling and solubility indices in the present study could be differences in the starch granule crystallinity. Viscosity patterns, the weak internal organization of starch, can also contribute to variations in solubility and swelling power of flour. The way in which amylose and amylopectin are distributed in the starch granule is thought to be another factor that greatly impacts the solubility index [44].

### 3.4. Microstructure Analysis of Green Banana Flour

#### 3.4.1. Scanning Electron Microscopy (SEM) of Starch Isolated from Green Banana Flour

**Figure 1:**
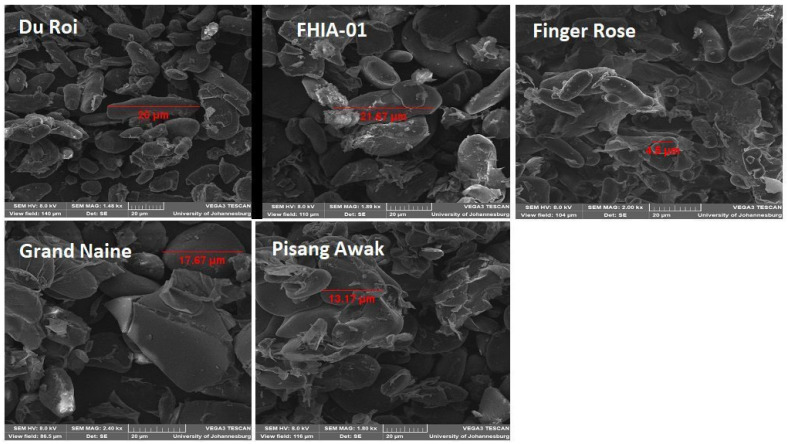
SEM micrographs green banana starch at 2000× Magnifications.

The SEM images of starch isolated from GBF are shown in Figure 1. SEM is used to study the surface morphology, structural integrity, as well as determinations of the size and shape of starch granules. In the present study, SEM revealed that starch granules from GBF were irregular in shape, and they had dense surfaces that had debris. The starch granules diameter ranged from 4.5 μm (Finger Rose) to 21.67 μm (FHIA-01). According to Reyes-Atrizco et al. [45] banana starch granules can vary from 4–35 μm in size, and this is in line with the size of GBF starch granules from this study. Finger Rose and Grand Naine exhibited longer, oval-shaped granules which had fragments on their surfaces. Du Roi granules were intact and elliptical in shape, while Pisang Awak and Finger Rose GBF had smaller and compact granules. The observed variation in GBF starch granules can affect the thermal property and swelling power. In the present study, samples with a bigger granule size had a higher water holding capacity. From the SEM images, it can be observed that the Finger Rose cultivar has the smallest starch granules compared to the other flours. Additionally, FHIA-01, Pisang Awak, Du Roi and Grande Naine had higher water holding capacities compared to Finger Rose, respectively. The SEM images of green banana flour show that there is a correlation between the flour morphology and water holding capacity.

According to Pandey et al. [46] the fragments that can be seen on the surface of the granules are probably amyloplast membranes which enclose starch granules in the banana fruit cell. The findings of the present study are akin to those by Reyes-Atrizco et al. [45] who reported that banana flour starch granules appear to be irregularly shaped, elongated and flattened, while the small granules are compact with spheroids and elongated forms.

#### 3.4.2. X-Ray Diffraction of Green Banana Flour

**Figure 2:**
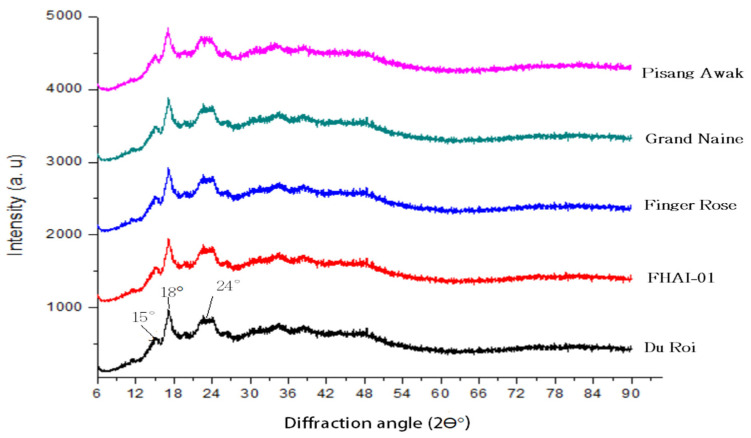
X-ray diffraction patterns of Pisang Awak, Grand Naine, Finger Rose, FHIA-01 and Du Roi.

There is a semi-crystalline nature of starch particles that can be assessed by XRD. In this study, the crystalline structure of green banana starch granules was analyzed using XRD, as shown in Figure 2. All GBF cultivars studied had similar diffraction patterns with prominent peaks at 15.00°, 18° and 24.00° diffraction angles. The GBF starch granules exhibited XRD patterns with three distinct peaks that were observed as a small peak at 15.00°, strong peak at 18.00°and a broad peak at 24. 00°. Generally, starch granules that originate from different sources appear to have varying crystallization characteristics. The three types of patterns that are displayed by starch are the A pattern (cereal starch), B (tuber, amylo-maize, and retrograded starch) pattern and C pattern (root and seed starches-pea and bean) [46,47,48]. In line with previous reports, the XRD pattern of green banana starch depicts the B-type crystallinity pattern irrespective of the variety and starch source [48].

#### 3.4.3. Fourier Transform Infrared Spectroscopy (FTIR)

**Figure 3:**
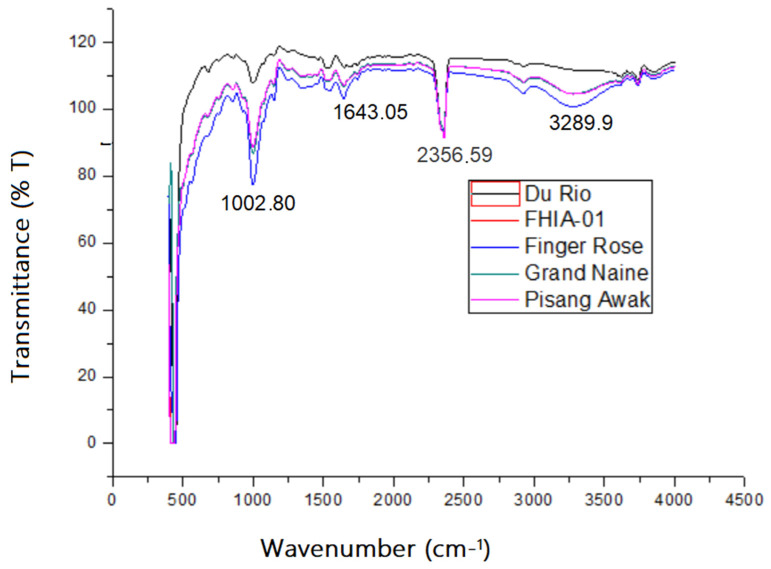
Comparative plots of the FTIR spectra of Du Roi, FHIA–01, Finger Rose, Grand Naine and Pisang Awak.

The FTIR analysis was performed to identify various characteristic functional groups present in the Grande Naine, Finger Rose, Du Roi and Pisang Awak green banana flours as shown in Figure 3. The identification of different functional groups in this study was conducted following the band/group assignments provided in the appendices section. For the Grand Naine, the absorption bands centered around 1643.05 cm^−1^ and 1002.80 cm^−1^ show the occurrence of hydroxyl (–OH), amine groups (–NH) and carbonyl group (=C=O) bonds, respectively [9]. The characteristic absorption bands of Grande Naine were similar to that of previous reports [9,47,49]. In general, the absorption bands between 800–1600 cm^−1^ are defined as the fingerprint region [49] There were characteristic bands of Finger Rose at 1002.80 cm^−1^, bands between 990 cm^−1^ and 1160 cm^−1^, attributed to carbonyl group (=C=O) bonds stretching. These compounds may contribute to the characteristic flavor and order of the banana flour. For all the analyzed banana flours, bands in similar regions were observed. Similar results have been reported elsewhere in the literature [20].

#### 3.4.4. In vitro Starch Digestion and Amylose Content of Green Banana Flour

Since humans generally consume cooked starch more than raw starch, the digestion performance of cooked banana starch is more important to the food industry. The digestibility fractions of green banana starch are indicated in Table 5. The GBF varied significantly (*p* ≤ 0.05) in their RDS, SDS and RS. Du Roi had the lowest RDS of 4.46%, while Grande Naine (6.02%) had the highest amount of RDS. The SDS ranged from 10.17% (FHIA-01) to 14.87% (Finger Rose). FHIA-01 had the highest amount of resistant starch (RS) (86.50%), while Grande Naine had the lowest amount of RS (80.38%). It is widely acknowledged that the GI and RS contents are two significant indicators of starch digestibility [12,50] These findings are an indication that GBF is a source of high RS, which could be linked with a lower GI. This suggests that diets that include GBF can positively influence blood glucose control and can potentially manage diabetes in patients. It is, however, worth noting that the GI of GBF may vary based on protein content, fat content, particle size and maturity and ripeness of the fruit [50]. In a study by Soto-Maldonado et al. [13] on the GI of whole banana and overripe banana pulp, it was observed that extended maturation resulted in an increase in GI, possibly due to a decrease in starch content. In the present study, the RS constituted the highest fraction in the green banana starch. These results are in agreement with the results previously reported for native banana starch (88.7%) and native plantain starch (85%) by Reyes-Atrizco et al. [45]. Recently, the health benefits of RS have been reported to be similar to those of dietary fiber when considering factors such as maintenance of gut homeostasis and promotion of the growth of beneficial gut microflora [50]. Thus, the GBF cultivars in this study may also be used in food applications as pre-biotics. Furthermore, RS is believed to control the amount of glucose released from starchy food, thus lowering the risk of obesity. Since starch is the most available carbohydrate in GBF, it must be noted that carbohydrates in food can influence processing characteristics and the development of designer foods [12].

There are several properties that are affected by how amylose and amylopectin are arranged in GBF. These include gelatinization, retrogradation as well as digestibility [25,51]. Here, a statistically significant (*p* < 0.05) variation in amylose content was observed, with the FHIA-01 cultivar showing the highest amylose content (24.82%) and Finger Rose recording the lowest amylose content (15.55%). It was also noted that the flours with high amylose content appeared to have high RS content. Thus, we propose that the amylose content could somewhat be positively correlated to the resistant starch. Flour with a higher amylose content is known to have a high solubility index since the amorphous region of starch granules primarily contains high amylose content [52,53]. Previous studies [54,55,56] suggest that a high amylose content of foods generally tends to give rise to a lower GI. The aforementioned was observed in the present study, as FHIA-01 had the highest amylose content and solubility index in comparison to the other studied GBF.

### 3.5. Phenolic Content and Antioxidant Activity of Green Banana Flour

#### 3.5.1. Total Phenolic Content and Total Flavonoid Content

The TPC of GBF cultivars studies here significantly (*p* < 0.05) varied (Table 4). Grand Naine had the highest TPC (524.87 mg GAE/100 g), while Du Roi had the lowest TPC (298.73 mg GAE/100 g). Phenolic compounds are essential secondary metabolites that are relatively high in bananas when compared to other fruits [57] They have been associated with health benefits that include the prevention of several diseases, such as diabetes, obesity and cardiovascular disease. The TPC content of Grand Naine in this study was six times higher than that reported by Anyasi and Mchau [49], possibly because of differences in the stage of ripening, the growth conditions and agricultural practices. Moreover, the extent of maturity has been reported to substantially affect the total phenolic content in green banana flour [1,24,56]. Passo et al. [58] reported that over-ripened banana flour had 52% less phenolic content than GBF, while ripe banana flour had 15–45% less phenolic content than GBF. Banana flour contains phenolic compounds such as catecholamines, phenolic acids and flavonoids [57,59]. Turola et al. [60] also reported the presence of gallic acid, catechin, epicatechin and myricetin3-O-rhamnosyl-glucoside in ripe and unripe banana flour cultivars. Furthermore, phenolic compounds can be used as food additives in the food industry to prevent lipid oxidation reactions in food formulations. With its high TPC, the Grand Naine cultivar has the potential for being used as a raw material in functional foods. The variation in total phenolic content observed in the present study may be attributed to genetic differences amongst the different banana flours. Bananas contain phenolic compounds such as catecholamines, phenolic acids and flavonoids [49]. The variation in total phenolic content observed in the present results may be attributed to genetic differences amongst different banana flours. Grand Naine recorded higher TFC (407.08 mg QE/100 g) among the flours studied, while Du Roi cultivar had the lowest TFC (287.40 mg QE/100 g). According to Hofmann et al. [24], green banana is abundant in TPC and contains various flavonoids.

#### 3.5.2. Antioxidant Activity (AOA) of Green Banana Flour

The antioxidant capacity of GBF is shown in Table 6. Among the cultivars, Grand Naine recorded the highest antioxidant activity (437. 22 and 474.23 mg TE/100 g d.w.) by both DPPH and FRAP assays, respectively. The second highest antioxidant activity was recorded with the Finger Rose cultivar and again with both assays. From the results obtained in this study, it is worth noting that cultivars with high TPC showed high antioxidant capacity. Therefore, the finding that Grand Naine was the richest in antioxidant activity was credited to its relative great quantity of phenolic compounds. Previous studies have shown that food with high antioxidants (e.g., carotenoids) can improve immunity in humans. Such a benefit has been linked to the reduction in the occurrence of diseases such as cancer, cardiovascular diseases and diabetes [9]. The health benefits associated with antioxidants are believed to be due to the vital role they play in impeding the initial stages of lipid peroxidation and scavenging singlet oxygen [48]. According to Turola Barbi et al. [61] there is a relationship between DPPH inhibition for plant materials and the TPC and TFC. This was observed through increases in DPPH that occur with an increase in the concentration of phenolic compounds or degree of hydroxylation of the phenolic compounds. Such is consistent with the fact that the antioxidant activity in plants is greatly associated with the phenolic fraction. Although not investigated here, it must be noted that different structures within the same plant contain different concentrations of phenolic compounds [45,55,62]. The FRAP assay is commonly used to study the antioxidant capacity of plant materials. In this study, all GBF samples showed a high correlation between the FRAP value and DPPH value. This can be attributed to the fact that both DPPH and FRAP assays generally follow the same mechanism [49]. High correlations between different antioxidant activity methods have also been reported by other researchers [56,57]. The above indicates that banana fruits with high antioxidant capacity could have a high value for their potential health-promoting benefits.

## 4. Conclusions

The varying functional and physicochemical properties of the GBF cultivars suggest that they can be utilized as raw materials in different food products. The morphological characteristics of the GBF starch and the fact that it appears to be linked to high WAC suggest their possible application in the development of edible films. The GBF were all found to contain relatively high RS, which makes them suitable for the development of low-GI food products. The high TPC, TFC and antioxidant activity in the Grand Naine cultivar suggests its possible use in health-promoting food products. Green banana flour cultivars, such as Pisang Awak, FHIA and Du Roi, indicate that they could be stable when added to food that is processed at a high temperature due to their high amylose content. While, on the other hand, Grand Naine and Finger Rose have low amylose content, which suggests a possible use in food products with low thermal characteristics.

## Figures and Tables

**Table 1:** Proximate analyses of green banana cultivars grown in South Africa.

	Parameters (g/100 g d.w.)				
Samples	Moisture	Ash	Fat	Protein	Carbohydrate
Grande Naine	10.50 ± 0.71 ^b^	3.50 ± 0.11 ^b^	0.52 ± 0.00 ^b^	3.60 ± 0.69 ^a^	81.88 ± 1.4 ^a^
Pisang Awak	9.50 ± 0.33 ^a^	2.50 ± 0.05 ^a^	0.85 ± 0.02 ^e^	4.12 ± 0.48 ^c^	83.03 ± 0.81 ^b^
Finger Rose	10.50 ± 0.84 ^b^	3.43 ± 0.31 ^b^	0.70 ± 0.71 ^d^	4.33 ± 0.30 ^d^	81.04 ± 0.76 ^a^
FHIA-01	9.40 ± 1.34 ^a^	3.50 ± 0.22 ^b^	0.68 ± 0.61 ^c^	3.63 ± 0.77 ^a^	84.82 ± 0.90 ^d^
Du Roi	9.50 ± 0.51 ^a^	2.46 ± 0.32 ^a^	0.42 ± 0.51 ^a^	3.81 ± 0.43 ^b^	83.81 ± 0.56 ^c^

Values with different alphabets in a column indicate significant differences (*p* < 0.05). g/100 g—gram per hundred grams; d.w.—dry weight basis. (*n* = 3).

**Table 2:** Essential minerals in green banana flour from different cultivars (mg/100 g dry weight).

Minerals	Grande Naine	FHIA-01	Finger Rose	Pisang Awak	Du Roi
Essential macro minerals					
Ca	18.38 ± 0.23 ^c^	10.50 ± 0.33 ^b^	8.70± 0.19 ^a^	28.25 ± 0.34 ^e^	19.68 ± 0.41 ^d^
Mg	100.10 ± 0.17 ^d^	118.15 ± 0.14 ^e^	82.10 ± 0.15 ^c^	35.85 ± 0.38 ^b^	32.4 ± 0.20 ^a^
K	934.7 ± 0.11 ^d^	1033.25 ± 0.15 ^e^	878.95 ± 0.31 ^c^	501.58 ± 0.22 ^b^	290.95 ± 0.35 ^a^
P	99.25 ± 0.40 ^e^	85.43 ± 0.12 ^d^	72.50 ± 0.41 ^c^	38.38 ± 0.21 ^b^	31.72 ± 0.38 ^a^
S	77.23 ± 0.11 ^d^	136.61 ± 0.21 ^e^	66.69 ± 0.51 ^c^	55.55 ± 0.21 ^a^	58.35 ± 0.12 ^b^
Essential trace minerals					
Zn	0.93 ± 0.21 ^e^	0.53 ± 0.48 ^b^	0.57 ± 0.30 ^c^	0.28 ± 0.33 ^d^	0.18 ± 0.43 ^a^
Cu	0.5 ± 1.40 ^d^	0.43 ± 0.31 ^c^	0.33 ± 0.22 ^b^	0.33 ± 0.30 ^b^	0.25 ± 0.26 ^a^
Fe	2.88 ± 0.21 ^e^	1.50 ± 0.27 ^c^	1.33 ± 0.31 ^a^	2.30 ± 0.44 ^d^	1.48 ± 0.42 ^b^
Mn	3.20 ± 0.41 ^e^	1.23 ± 0.29 ^d^	0.98 ± 0.25 ^c^	0.60 ± 0.21 ^b^	0.48 ± 0.11 ^a^

Values with different alphabets in a column indicate significant differences (*p* < 0.05). d.w.—dry weight basis (*n* = 3).

**Table 3:** The water absorption capacity of green banana flour cultivars.

Banana Flour Samples	Water Absorption Capacity (%)
FHIA-01	58.01 ± 0.31 ^d^
Grande Naine	43.18 ± 0.10 ^b^
Pisang Awak	67.11 ± 0.00 ^e^
Finger Rose	40.00 ± 0.58 ^a^
Du Roi	50.12 ± 0.69 ^c^

Values with different alphabets in a column indicate significant differences (*p* < 0.05). The water absorption capacity was expressed on a dry weight basis (d.w.). (*n* = 3)

**Table 4:** The water solubility and swelling index of banana flour (d.w.).

Banana Flour Samples	Solubility (%)			Swelling Power (g/g)		
	50 °C	70 °C	90 °C	50 °C	70 °C	90 °C
FHIA-01	6.49 ± 0.73 ^b^	9.50 ± 0.71 ^d^	15.01 ± 0.71 ^d^	0.29 ± 0.71 ^a^	0.42 ± 0.71 ^a^	0.52 ± 0.95 ^a^
Grande Naine	7.40 ± 0.00 ^c^	9.61 ± 0.34 ^d^	10.21 ± 0.59 ^b^	0.50 ± 0.19 ^d^	0.67 ± 0.71 ^b^	0.75 ± 0.00 ^b^
Pisang Awak	6.50 ± 0.32 ^b^	8.47 ± 0.58 ^b^	11.4 ± 0.58 ^c^	0.33 ± 0.21 ^b^	0.38± 0.44 ^a^	0.53 ± 0.01 ^a^
Finger Rose	7.0 ± 0.08 ^c^	9.01 ± 0.34 ^c^	10.21 ± 0.59 ^b^	0.41± 0.79 ^c^	0.67 ± 0.71 ^b^	0.79 ± 0.04 ^b^
Du Roi	5.50 ± 0.71 ^a^	7.59 ± 0.06 ^a^	8.03 ± 0.53 ^a^	0.38 ± 0.24 ^c^	0.63 ± 0.27 ^b^	0.83 ± 0.54 ^c^

Values with different alphabets in a column indicate significant differences (*p* < 0.05). The results are expressed on a dry weight basis (d.w.). g/g—gram per gram. (*n* = 3).

**Table 5:** Rapidly digestible starch (RDS), slowly digestible starch (SDS), resistant starch (RS) and amylose content green banana flour.

Samples	RDS (%)	SDS (%)	RS (%)	Amylose (%)
Grande Naine	6.02 ± 0.11 ^c^	13.30 ± 0.00 ^d^	80.38 ± 1.41 ^a^	18.95 ± 0.98 ^b^
Pisang Awak	5.50 ± 0.05 ^b^	11.73 ± 0.02 ^c^	84.35 ± 1.51 ^c^	23.00 ± 0.91 ^d^
Finger Rose	5.43 ± 0.31 ^b^	14.87 ± 0.01 ^e^	81.70 ± 1.21 ^b^	15.55 ± 0.90 ^a^
FHIA-01	4.50 ± 0.22 ^a^	10.17 ± 0.61 ^a^	86.50 ± 0.21 ^e^	24.82 ± 0.00 ^e^
Du Roi	4.46 ± 0.82 ^a^	10.42 ± 0.51 ^b^	85.50 ± 0.40 ^d^	21.32 ± 0.16 ^c^

Values with different alphabets in a column indicate significant differences (*p* < 0.05). (*n* = 3).

**Table 6:** Total flavonoid content (TFC), total phenolic content (TPC) of green banana flour.

Samples	TPC (mg GAE/100 g d.w.)	TFC (mg QE/100 g d.w.)	DPPH (mg TE/100 g d.w.)	FRAP (mg TE/100 g d.w.)
FHIA-01	307.03 ± 0.7 ^b^	293.87 ± 0.91 ^b^	359.11 ± 0.7 ^b^	411.72 ± 0.7 ^b^
Grande Naine	524.87 ± 1.6 ^d^	407.08 ± 1.7 ^d^	437. 22 ± 1.0 ^c^	474.23 ± 0.2 ^c^
Pisang Awak	312.00 ± 2.1 ^b^	291.80 ± 0.9 ^b^	363. 28 ± 0.4 ^b^	397. 11 ± 1.1 ^b^
Finger rose	321.87 ± 0.1 ^c^	305.01 ± 1.0 ^c^	421.00 ± 0.1 ^c^	448.87 ± 1.3 ^c^
Du Roi	298.73 ± 1.1 ^a^	287.40 ± 2.1 ^a^	301.34 ± 1.1 ^a^	324.27 ± 0.1 ^a^

Values with different alphabets in a column indicate significant differences (*p* < 0.05). d.w.–dry weight. (*n* = 3).

## Data Availability

Not applicable.
